# Comprehensive analysis based on glycolytic and glutaminolytic pathways signature for predicting prognosis and immunotherapy in ovarian cancer

**DOI:** 10.7150/jca.88359

**Published:** 2024-01-01

**Authors:** Zihui Zhang, Yuqin Huang, Shuang Li, Li Hong

**Affiliations:** 1 Department of Gynecology and Obstetrics, Renmin Hospital of Wuhan University, Wuhan, Hubei Province, People's Republic of China.; 2 Department of Gynecology and Obstetrics, Xiangyang No. 1 People's Hospital, Hubei University of Medicine, Xiangyang, Hubei Province, People's Republic of China.

**Keywords:** Aerobic glycolysis, Glutaminolytic, Ovarian cancer, Prognosis, Immunotherapy.

## Abstract

**Background:** Our study attempts to develop and identify an aerobic glycolysis and glutamine-related genes (AGGRGs) signature for estimating prognostic effectively of ovarian cancer (OV) patients.

**Materials & methods:** OV related data were extracted from the multiple public databases, including TCGA-OV, GSE26193, GSE63885, and ICGC-OV. A consistent clustering approach was used to characterize the subtypes associated with AGGRGs. LASSO Cox regressions was utilized to construct the prognosis signatures of AGGRGs. In addition, GSE26193, GSE63885 and ICGC-OV served as independent external cohorts to assess the reliability of the model. *In vitro and in vivo* experiments were conducted to study the role of AAK1 in the malignant progression and glutamine metabolism of OV, and assessed its therapeutic potential for treating OV patients.

**Results:** OV patients could be separated into four subtypes (quiescent, glycolysis, glutaminolytic, and mixed subtypes). The survival outcome of glutaminolytic subtype was notably worse than the glycolytic subtype. Besides, we identified eight AGGRGs (AAK1, GJB6, HMGN5, LPIN3, INTS6L, PPOX, SPAG4, and ZNF316) to establish a prognostic signature for OV patients. Comprehensive analysis revealed that the signature risk score served as an independent prognostic factor for OV. Additionally, high-risk OV patients were less sensitive to platinum and, conversely, were proved to be more responsive to immunotherapy than low-risk score. In cytological experiments, we found that AAK1 could promote cancer progression and glutamine metabolism via activating the Notch3 pathway in OV cells. Furthermore, knockdown of AAK1 significantly inhibited tumor growth and weight, decreased lung metastases, and ultimately extended the survival time of the nude mice.

**Conclusions:** The prognostic signature of AGGRGs constructed could efficiently estimate the prognosis and immunotherapy effectiveness of OV patients. In addition, AAK1 may represent a promising therapeutic target for OV.

## Introduction

Ovarian cancer (OV) is widely acknowledged as the most lethal gynecologic malignancy. According to literature reports, the global incidence of OV in 2020 was recorded as 313,959 patients with a total mortality count of 207,252 patients 1. Although treatments such as surgery and platinum-based chemotherapy have become standard, five-year survival rates for OV patients remain below 50 percent, mainly due to the challenges of recurrence and chemotherapy resistance 2. Correspondingly, the investigation of potential therapeutic targets, as well as diagnostic or prognostic factors, is crucial for OV patients. A considerable body of evidence suggested that the metabolic pathways of glycogen, lipids, amino acids, and other substances were strongly associated with the diagnosis, chemotherapies, and prognosis of OV 3-5. In this regard, metabolism provided a promising target to combat cancer progression and assess prognosis.

A previous study has proposed that aerobic glycolysis, an important feature of tumor metabolic reprogramming, contributes to malignancy progression, chemotherapy resistance, and immune evasion 6. For instance, mitochondrial calcium uptake 1 (MICU1) was associated with aerobic glycolysis and chemotherapy resistance in OV 7. Tankyrase activation of the Wnt/β-Catenin signal pathway and aerobic glycolysis facilitated the malignant progression 8. Importantly, genes associated with glycolysis were effective predictors of survival in cases of OV 9. While studies have indicated that aerobic glycolysis was the primary means for tumor cells to produce energy, the dysregulation of fatty acids and amino acids, particularly glutamine, serine and glycine, through synthesis/catabolism pathways, were also significantly associated with tumor energy metabolism 10, 11. Recent research has revealed that the primary energy source for cancer cells was glutamine, rather than glucose 12. For instance, glutamine participated in the progression of OV as well as in the development of resistance to chemotherapy 13. Glutamine metabolism was significantly increased in platinum-resistant OV cells 14. One possible critical mechanism was that the oncogene MYC promoted platinum resistance by up-regulating glutaminase (GLS) 15. Additionally, Glutamate-ammonia ligase (GLUL), popularly known as glutamine synthetase (GS), was extremely expressed in OV and associated with poor prognosis 16. Thus, metabolic reprogramming, especially aerobic glycolysis and glutamine metabolism, were strongly associated with patient outcomes. Regrettably, at present, there is a lack of comprehensive assessment regarding the prognostic of glycolysis and glutamine metabolism in forecasting the survival of OV patients.

**Figure 1:**
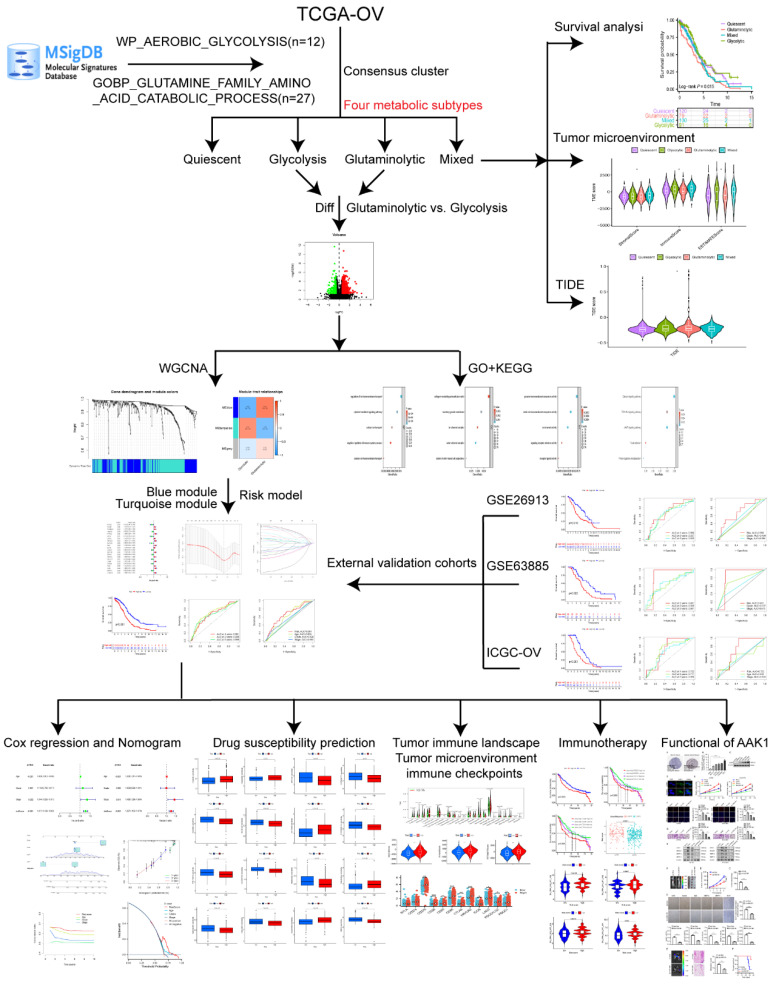
The workflow of this study.

Considering the significant roles of aerobic glycolysis and glutamine genes in OV, we aimed to classify the OV patients into four distinct metabolic subtypes according to the two metabolic gene expressions. In addition, we developed a prognosis model for prognostic stratification and drug efficacy prediction depended on the aerobic glycolysis and glutamine-related genes (AGGRGs), and examined the relationship of tumor microenvironment and immune infiltration. The process of this research was indicated in **Figure 1.**

## Methods

### Data collection and processing

TCGA-OV and GTEx data were extracted from the UCSC XENA (https://xenabrowser.net/datapages/) by the Toil process 17, which integrates TCGA-GTEx and TPM formats. Moreover, three datasets were served as external validation cohorts, including GSE26193 18, GSE63885 19, ICGC-OV (https://dcc.icgc.org/). The relevant clinical features of those datasets were shown in **Table 1.** Aerobic glycolytic pathway genes (WP_AEROBIC_GLYCOLYSIS, n = 12) and the glutaminolytic pathway genes (GOBP_GLUTAMINE_FAMILY_AMINO_ACID_CATABOLIC_PROCESS, n = 27) were collected from the MSigDB database 20 (**Table S1**).

### Identification of the AGGRGs-related subtypes

According to previous study 21, the “ConsensusClusterPlus” R package was utilized to perform consensus clustering of OV samples. Ultimately, according to the median expression level of co-expressed metabolic genes, we classified the OV patients into four completely distinct metabolic subtypes: quiescent type, glycolytic type, glutaminolytic type, and mixed type.

### Weighted gene co-expression network analysis (WGCNA) and enrichment analysis

Firstly, the "limma" package was applied to discover the dysregulated genes between the glutaminolytic type and glycolytic type according to | Log_2_FC | > 0.585 and *P*. adj < 0.05 standard. Next, consistent with the approach that Zhang et al. has adopted 21, the "WGCNA" package was applied to pinpoint the key genes associated with metabolic variances between the glutaminolytic and glycolytic subtypes. The "ClusterProfiler" package was employed to conduct Gene Ontology (GO) and Kyoto Encyclopedia of Genes and Genomes (KEGG) analysis of the key metabolic genes.

### Establishment and validation of an AGGRGs prognostic signature

An AGGRGs prognostic model was conducted by utilizing univariate Cox, LASSO, and multivariate Cox regressions established on the TCGA-OV dataset. A risk score was computed for each sample utilizing the following algorithm: Riskscore = Σ coef × expression (coefficient, coef). The samples were separated into low- or high-risk founded on the median risk score of the constructed model. Additionally, the samples were haphazardly separated into training and testing cohorts at a 1:1 ratio. Moreover, survival analysis as well as receiver operating characteristic (ROC) curve analysis were performed to assess the effectiveness of the model across multiple cohorts, including the TCGA-OV train cohort (n = 210), TCGA-OV test cohort (n = 210), TCGA-OV all cohort (n = 420), as well as the GSE26193 (n = 107), GSE63885 (n = 75), and ICGC-OV (n = 111) cohorts. In addition, a nomogram was established to forecast the 1-, 3-, and 5-year overall survival (OS) rates for OV. We evaluated the discriminative ability of the model using a calibration curve and a concordance index (C-index). Additionally, the clinical outcomes of various decision-making strategies were evaluated by decision curve analysis (DCA).

### Drug sensitivity prediction and immune landscape

The theoretical basis and reference for individualized treatment of clinical patients were evaluated by comparing drug sensitivity using the "oncoPredict" package 22, which predicts the half maximal inhibitory concentration (IC50) of potential drugs. Besides, the CIBERSORT algorithm was used to compare levels of immune cell infiltration among low- and high-risk groups.

### Evaluation of the Immunotherapy

Firstly, the risk score with each patient in the IMvigor210 cohort was quantified based on the previous risk algorithm, and subsequently performed survival analysis and immunotherapy prediction. We next explored the immune efficacy of targeting CTLA-4 and PD-1 relying on the TCIA dataset (The Cancer Immunome Atlas, https://tcia.at/home).

### Cell culture

Normal human ovarian epithelial cells IOSE-80, and multiple OV cell lines including SKOV3, A2780, OVCAR3, OVCAR8, and ES-2 were obtained from the China Center for Type Culture Collection (Hubei, China). IOSE-80, SKOV3, A2780 were grown in RPMI-1640 medium, ES-2 was grown in McCoy's 5A medium, and OVCAR3, OVCAR8 were cultured in DMEM medium. All medium applied for cell culture were added with 1% penicillin-streptomycin mixture and 10% fetal bovine serum. All cell lines were cultured at 5% CO2, and 95% air at 37 °C.

### CCK-8 assay

2 × 10^3^ cells per well were inoculated into 96-well plates and cultured for the indicated times. Then 10 μl of CCK-8 solution (40203ES76, Yeasen Biotechnology Co., Ltd, Shanghai, China) was infused into each well and were incubated for 2h. The optical density (OD) of each chamber was calculated at 450 nm utilizing a microplate reader (EnSight, USA) and cell viability was quantified.

### EdU assay

Cells planted into 24-well plates (5 × 10^4^ cells per well) were cultured at 80% confluence by using EdU Imaging Kit (C0075S, Beyotime Institute of Biotechnology, Jiangsu, China). In a nutshell, cells in each chamber were marked with 10 μM EdU solution for 2 h. Next, cells were immobilized utilizing 4% paraformaldehyde for 10 min, then the nucleus was stained 5 min by DAPI in the dark. Photographs were obtained using a fluorescence microscope (Olympus, Tokyo, Japan). The Image J software (v.1.8.0) was utilized to assess the number of EdU-positive cells.

### Transwell invasion assay

Matrigel invasion assay was executed to determine the cell invasion capability as previously published 23. The result was calculated utilizing Image J software (v.1.8.0).

### Intracellular glutamate (Glu), α-Ketoglutaric Acid (α-KG) and ATP levels

The intracellular levels of glutamate, α-KG, and ATP were measured using the Glutamate Content Assay Kit (Sosarbio, BC1585), α-KG Content Assay Kit (Sosarbio, BC5425), and ATP Colorimetric Assay Kit (BioVision, EATP-100), according to the manufacturer's instructions.

### Reduced glutathione (GSH) and reactive oxygen species (ROS) assay

The intracellular concentration of GSH was assessed utilizing the GSH Assay Kit (Nanjing Jiancheng Bioengineering Institute, A006-2-1) based on the instructions provided by the manufacturer. Dihydroethidium Assay Kit (Beyotime, S0063) was used to detect intracellular ROS levels. The observations were made on an Olympus IX71 fluorescence microscope in a blinded manner and the average fluorescence intensity was determined by analyzing the images using image J software (v.1.8.0).

### Gene over-expression and knockdown

Human AAK1(Gene id: 22848) overexpression vectors, and an empty vector containing pGPU6 were acquired from Ribobio (Guangzhou, China). The shRNA sequences against AAK1 (sh-AAK1 #1 and sh-AAK1 #2) were obtained from RiboBio (Guangzhou, China) with pGPU6 vector. The AAK1 shRNA sequences were presented as follows: shRNA#1: GGCTGAAGATGAGTTTGACCCTATT; shRNA#2: GAGCACCAGAAATGGTCAACCTGTA.

### Real-time quantitative PCR (RT-qPCR)

RT-qPCR was conducted following the established protocol as previously described 23. PCR primers were listed below: AAK1 (forward): AGTTTGCCCCCATAGCACTC, (reverse): CCTAGAGTGCCCACCTTGTG. β-actin (forward): CGCGGCGATATCATCATCCA, (reverse): CGGCTTCCTTTGTCCCCAAT.

### Western blotting

Western blotting assay was executed as previously reported 23, with rabbit anti-human antibodies for AAK1 (1:2000, PA5408, Abmart), Notch3 (1:1000, PS08936, Abmart), GLS (1:2000, T55719, Abmart), MMP-2 (1:1000, PA1748, Abmart), MMP-9 (1:2000, TA5228, Abmart), and β-actin (1:2000, GB11001, Servicebio).

### Immunofluorescence staining

In brief, after formalin fixing, cells were permeabilized with 0.1% Triton X-100 in TBS for 5 minutes, followed by a 30-minute block with goat serum. Next, cells were subjected to incubation with the AAK1 antibody (1:50, PA5408, Abmart) for 1h and hatched with FITC-conjugated goat anti-rabbit IgG (1:100, GB22303, servicebio) at room temperature for 2 hours at 37°C. Subsequently, the nucleus was marked with DAPI (BL105A, Biosharp). Finally, the cells were captured using a fluorescence microscope (Olympus, Tokyo, Japan).

### Hematoxylin and eosin (HE) staining and immunohistochemistry (IHC) staining

The HE, IHC protocol and result analysis method were performed as previously described 24, with rabbit anti-human antibodies for AAK1 (1:200, PA5408, Abmart), Notch3 (1:10, PS08936, Abmart), GLS (1:100, T55719, Abmart), MMP-2 (1:100, PA1748, Abmart), MMP-9 (1:300, TA5228, Abmart), and Ki-67 (1:200, GB111141, Servicebio).

### Subcutaneous graft tumor and lung metastasis model

Briefly, 4 weeks old female BALB/c nude mice, were injected with 1 × 10^6^ cells that had been stably transfected into the right dorsal flank (n = 5 per group). Tumor diameters were measured weekly using a caliper, and the volume was calculated as follows: tumor volume = length × (width)²/2.

For the tumor lung metastasis assay, 1 × 10^7^ cells were injected into the tail veins of male BALB/c nude mice that were 4 weeks old (n = 5 per group). The lung tissues were performed to HE staining, and the number of lung metastatic nodules was randomly counted.

All experimental animal protocols adhered to the NIH Guidelines for the Care and Use of Laboratory Animals and received approval from the Animal Research Ethics Committee of the Renmin Hospital of Wuhan University.

### Statistical analysis

Statistical analysis was achieved in R software (version 4.0.2) and GraphPad Prism (version 8.0). The student's *t*-test was utilized to compare data between the two independent groups. One-way analysis of variance (ANOVA) with Bonferroni's correction was used to analyze the comparisons among multiple groups. Log-rank test was applied to survival analysis. The statistical significance was deemed significant when the* P* < 0.05.

## Results

### Identification of the four metabolic subtypes of OV

**Figure 2:**
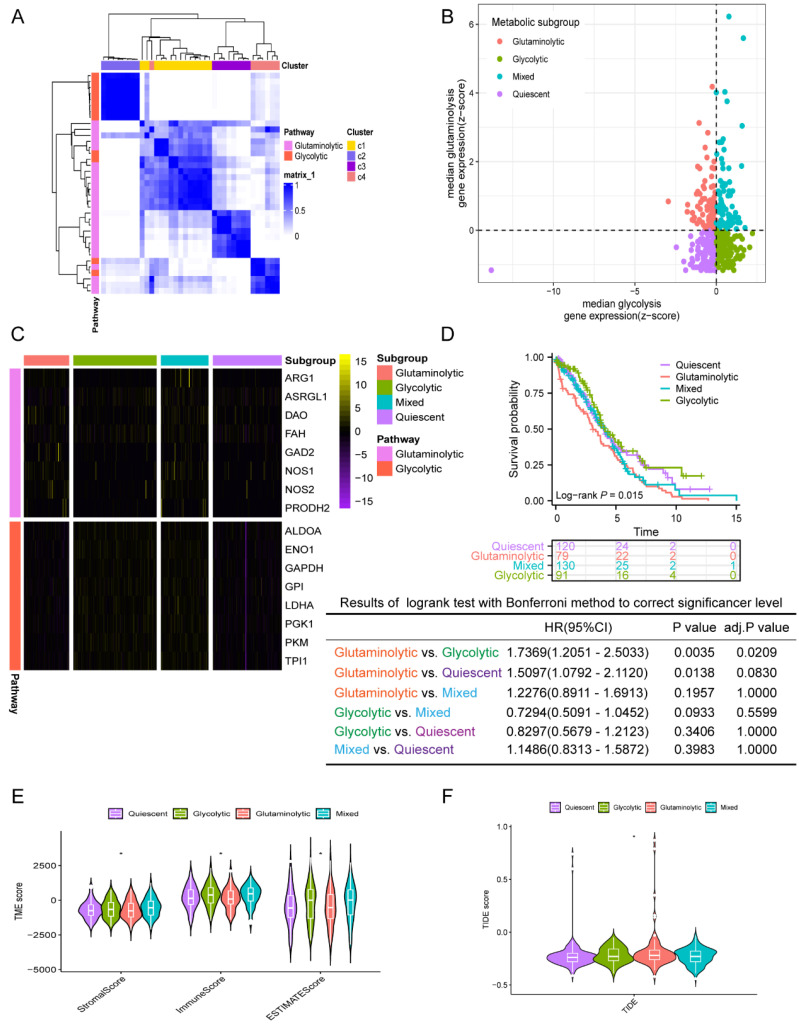
** Classification of the four metabolic subtypes based on the expression of AGGRGs in OV patients.** (**A**) Consistent clustering of the AGGRGs based on TCGA-OV samples. (**B**) Scatter plot revealing the four metabolic subtypes based on AGGRGs expression. (**C**) Heatmap indicating the co-expressed AGGRGs levels in the four metabolic subtypes. (**D**) Comparison of the survival curves among the four metabolic subtypes. (**E-F**) Violin plot displaying the immune score, stromal score, ESTIMATE score and TIDE score among the four metabolic subtypes, respectively. * *P* < 0.05, ** *P* < 0.01, ***P < 0.001.

First, the consensuscluster classification of AGGRGs was executed utilizing the "ConsensusClusterPlus" R package grounded on the TCGA-OV dataset, and the data suggested that when K=4, the glycolytic and glutaminolytic genes were collected in a cluster, respectively. As revealed in **Figure 2A**, the co-expressed genes in C2 (characterized as glycolytic genes, including ALDOA, ENO1, GAPDH, GPI, LDHA, PGK1, PKM, TPI1) be categorized as the glycolytic metabolic pathway, and the co-expressed genes in C3 (characterized as glutaminolytic genes, including ARG1, ASRGL1, DAO, FAH, GAD2, NOS1, NOS2, PRODH2) belong to the glutaminolytic metabolic pathway. Patients were delineated into four metabolic subtypes based on the two types of co-expressed metabolism gene sets, as described approach in the earlier literature 21 (**Figure 2B**), including quiescent subtype, glycolytic subtype, glutaminolytic subtype, and mixed subtype.

The gene expression levels between the four metabolic subtypes were shown in** Figure 2C**. Furthermore, an analysis of the prognostic value between the four subtypes revealed significant differences among them. As shown in **Figure 2D**, the OS of glutaminolytic subtype was extremely worse than the glycolytic subtype. Prior research has demonstrated that metabolic reprogramming related intimately to changes in the tumor microenvironment and malignancy progression 25, therefore, we explored the difference in tumor microenvironment across the four metabolic subtypes. The immune, stromal, and ESTIMATE scores of the four metabolic subtypes were from high to low: mixed subtype, glycolysis subtype, quiescent subtype, glutaminolytic subtype (**Figure 2E**). Furthermore, we evaluated the feasibility of immunotherapy in four metabolic subtypes based on the Tumor Immune Dysfunction and Exclusion (TIDE) algorithm (http://tide.dfci.harvard.edu) 26. The analysis revealed that the TIDE score was notably elevated in the glutaminolytic subtype compared to all other subtypes (**Figure 2F**), which indicated that the glutaminolytic subtype responds worse to immunotherapy than the other subtypes. In brief, our results suggested that the four metabolic subtypes could effectively assess the prognosis and efficacy of immunotherapy based on the glycolytic and glutaminolytic genes in OV.

### Identification of co-expression network associated with glutaminolytic-glycolytic types

To further uncover the difference genes, we performed a differential expression analysis. Among these, a total of 750 differentially expressed genes (DEGs) were discovered, including 218 decreased expression genes and 532 increased expression genes in the glutaminolytic subtype (**Figure S1A**). Subsequently, we conducted a WGCNA depending on the 750 differentially expressed genes (**Figure S1B**). To identify distinct gene modules, the dynamic cutting method was utilized. The modules were further filtered by employing a soft threshold of 0.8, resulting in the identification of three different gene modules: turquoise, blue, and grey (**Figure S1C-E**). In order to screen modules with significant correlation to glycolysis and glutamine subtypes, the eigenvalue (ME) of each module was calculated, respectively. The correlation heat map was displayed in **Figure S1F**, and the blue module suggested a positive correlation with the glutaminolytic subtype (*R* = 0.75, *P* = 6E-36), while the turquoise module was significantly correlated with the glycolytic subtype (*R* = 070, *P* = 3.2E-41) (**Figure S1F**). Next, the GO enrichment analysis unveiled that the biological process (BP) category was predominantly enriched in the regulation of ion transmembrane transport, cytokine-mediated signaling pathway, calcium ion transport, negative regulation of immune system process, and calcium ion transmembrane transport (**Figure S1G**). Besides, cell composition (CC) was primarily enriched in collagen-containing extracellular matrix, secretory granule membrane, ion channel complex, cation channel complex, and cluster of actin-based cell projections (**Figure S1H**). Furthermore, molecular function (MF) was mostly enriched in passive transmembrane transporter activity, metal ion transmembrane transporter activity, ion channel activity, signaling receptor activator activity, and receptor ligand activity (**Figure S1I**). Those findings demonstrated that the metabolism-related genes were widely involved in the complex and diverse cellular biological processes of OV. In addition, highly enriched KEGG pathway indicated calcium signaling pathway, PI3K-Akt signaling pathway, cAMP signaling pathway, focal adhesion, and protein digestion and absorption (**Figure S1J**). These metabolic-related pathways might be closely related to malignant progression 27, immune escape 28 and chemotherapy resistance 29.

### Construction and verification of a prognostic model based on AGGRGs signature

**Figure 3:**
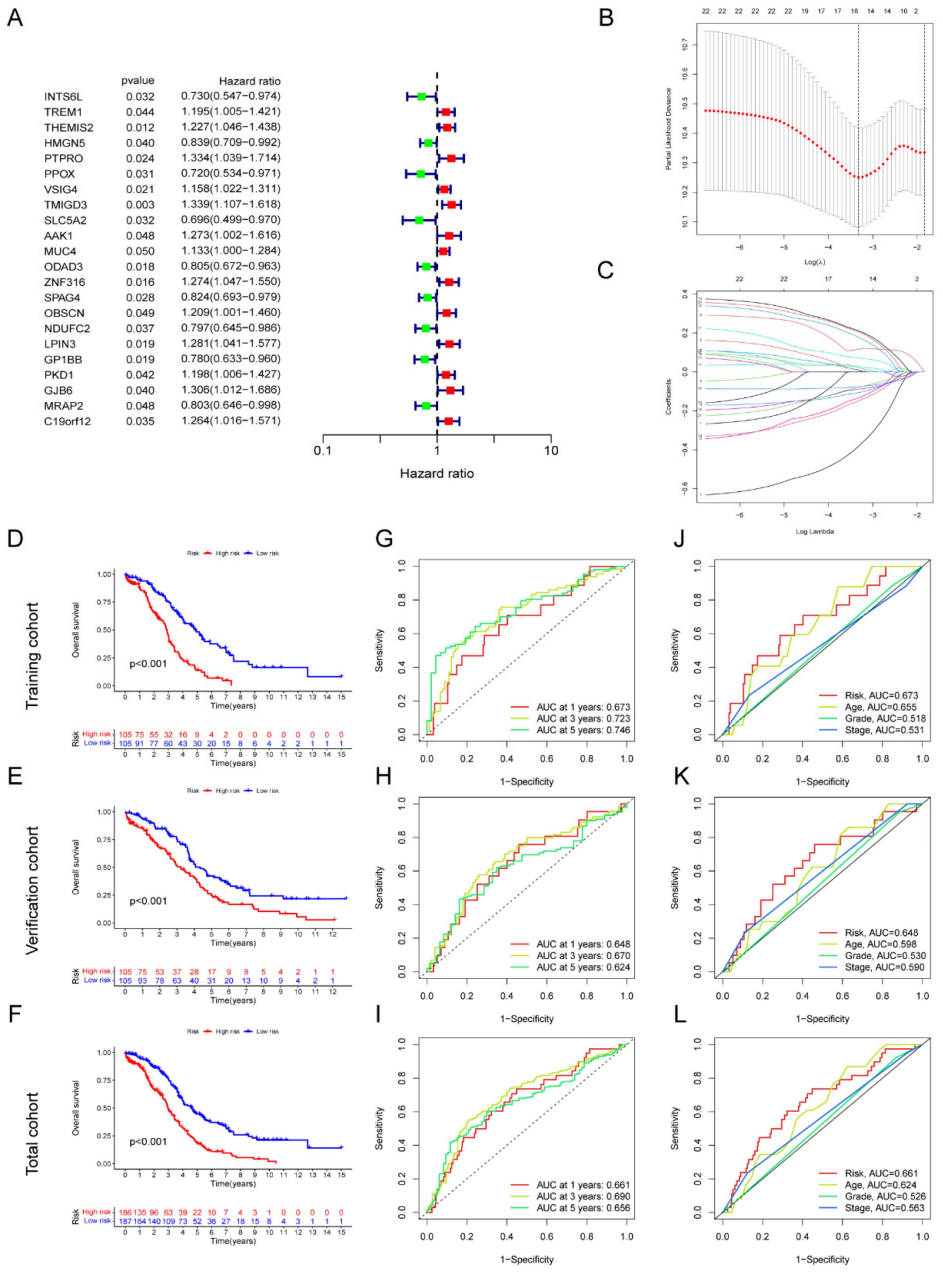
** Development of AGGRGs prognostic model established on TCGA-OV dataset.** (**A**) Univariate Cox analysis of the prognostic-related genes. (**B-C**) LASSO regression was applied to determine candidate prognostic-related genes. (**D-F**) Represent the K-M plot between the low- and high-risk groups in the training cohort (**D**), test cohort (**E**), and total cohort (**F**), respectively. (**G-I**) ROC curves for the prognostic capability of prognostic model in the training cohort (**G**), test cohort (**H**), and total cohort (**I**), respectively. (**J-L**) The ROC curves of the risk score and other clinical indicators for OS in training cohort, verification cohort, total cohort, respectively.

In total, 22 genes correlated with prognosis were selected based on the metabolic-related genes (containing genes in the blue and turquoise modules) applying univariate Cox regression, including 13 genes with high-risk (HR > 1) and 9 genes with low-risk (HR < 1) (**Figure 3A**). Moreover, we identified eight key metabolic genes suitable for the construction of prognostic model by using LASSO and multivariate Cox regressions (**Figure 3B-C**). The risk score for each patient was computed by taking into account the gene expression levels and regression coefficients. Risk score = (0.219436774166723 ×AAK1 expression) + (0.318685645443347 × GJB6 expression) + (-0.202206865106937 × HMGN5 expression) + (0.210947750407656 × LPIN3 expression) + (-0.56026035245543 × INTS6L expression) + (-0.356089292934913 × PPOX expression) + (-0.298411359156902 × SPAG4 expression) + (0.421629173986513 × ZNF316 expression). The total cohort of 420 samples was randomly classified into two cohorts: a training cohort (n=210) and a test cohort (n=210). The OS analysis demonstrated that high-risk groups within the training, test, and total cohorts consistently had poor outcomes (*P* < 0.05) (**Figure 3D-F**). The area under the curve (AUC) value exceeds 0.6 across all three cohorts at 1-, 3-, and 5-years (**Figure 3G-I**). Next, we assessed the risk scores in conjunction with clinicopathological indicators and observed that the AUC of the risk score outperformed other clinical indicators in terms of accuracy across all three groups (**Figure 3J-L**). Additionally, high-risk patients with age > 60, G3-4, and stage Ⅲ-Ⅳ had worse prognosis in three cohorts (*P* < 0.05) (**Figure S2**). Furthermore, the reliability of the model was verified in multiple independent external cohorts, including GSE26913, GSE63885, and ICGC-OV (**Figure S3**).

**Figure 4:**
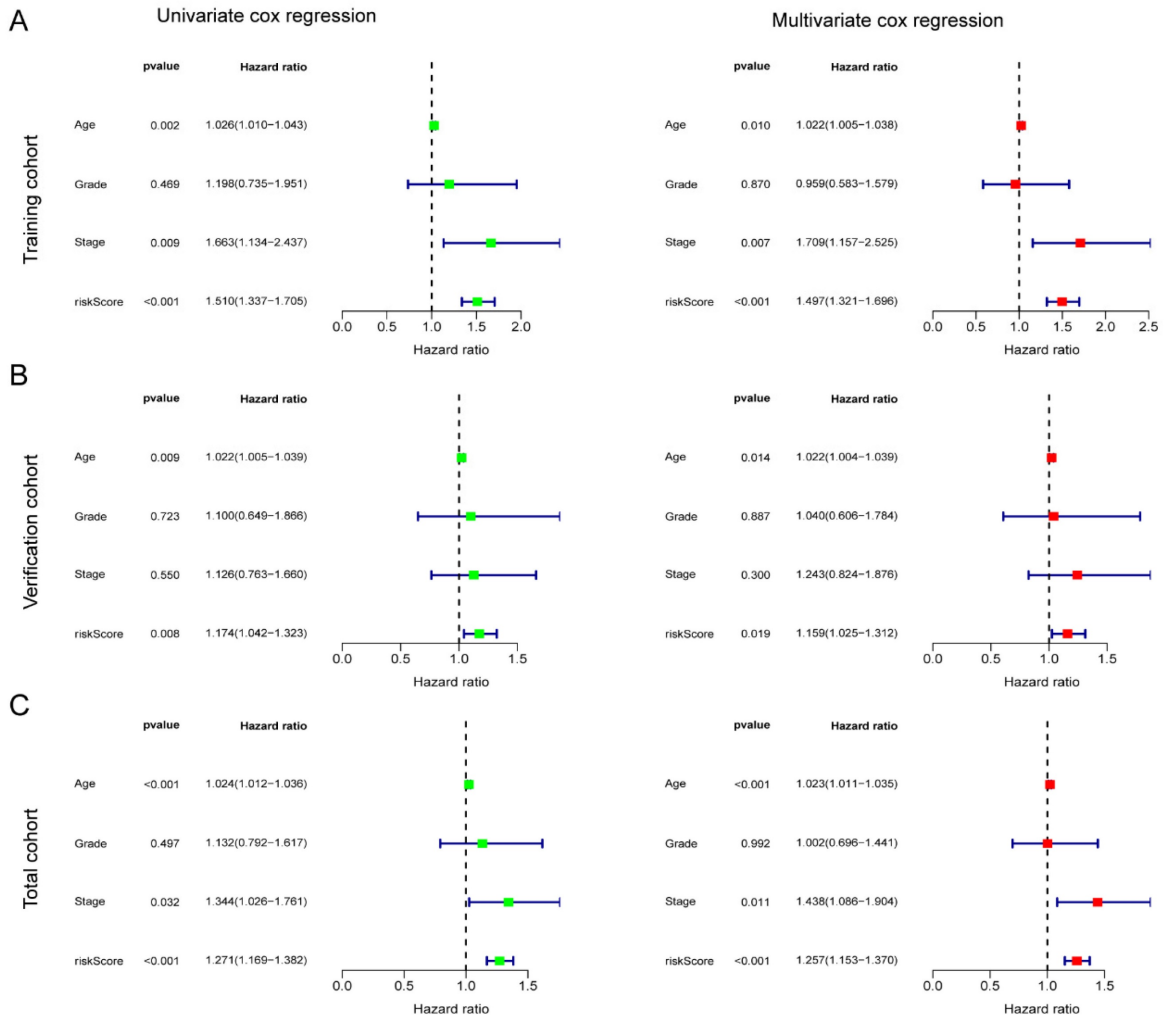
** Independent prognostic significance of the AGGRGs risk model.** (**A-C**) Univariate cox and multivariate cox regression analysis in training cohort (**A**), verification cohort (**B**), and total cohort (**C**), respectively.

**Figure 5:**
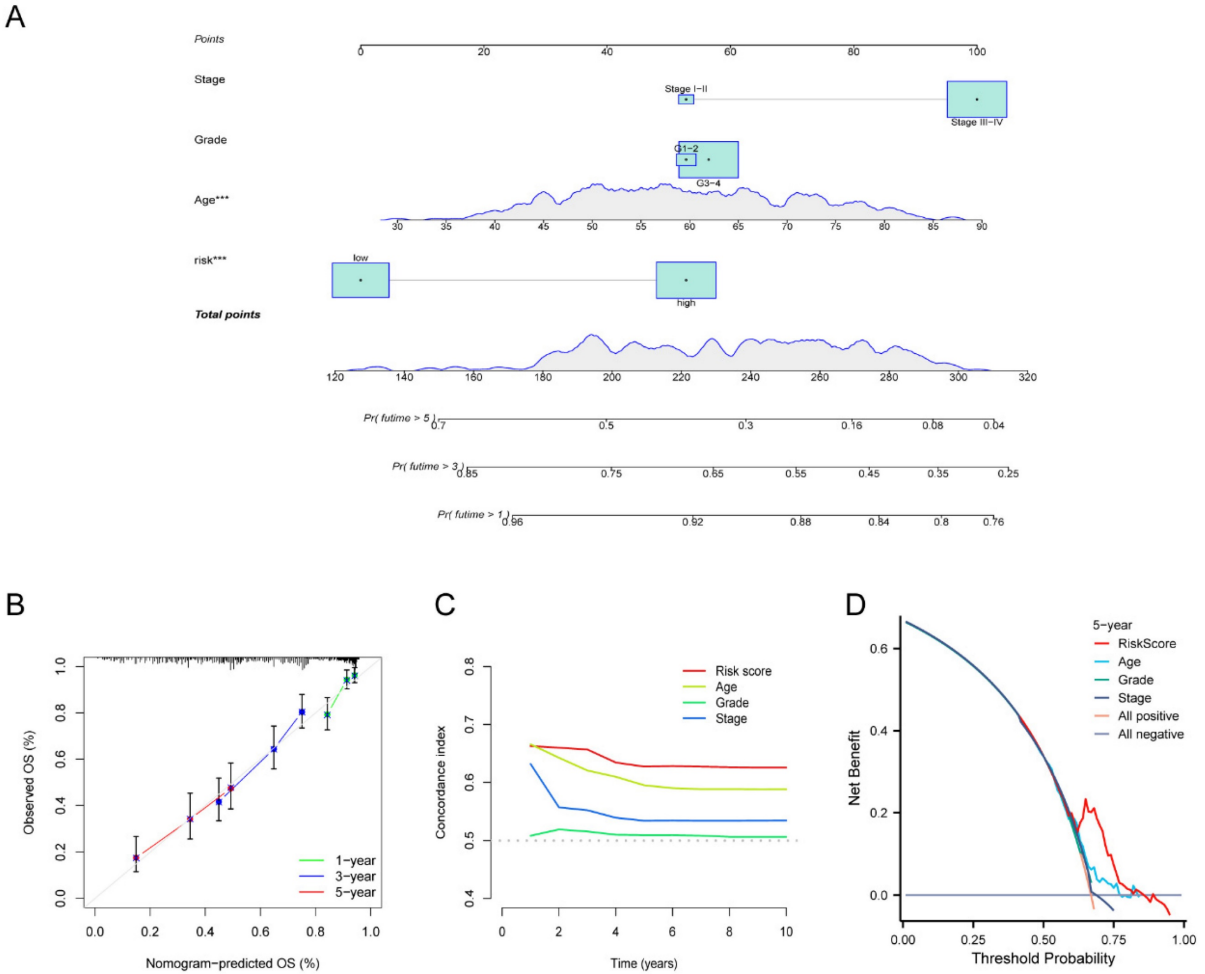
** Development and validation of the nomogram.** (**A**) ​Nomogram to forecast the survival rate of OV at 1-, 3-, and 5-year. (**B-C**) Calibration curve (**B**) and concordance index (C-index) (**C**) revealed the predictive capacity and reliability of AGGRGs prognostic model. (**D**) The DCA diagram evaluated the clinical factor of the risk model.

To verify the independence of the risk score from other clinical indicators, Cox regression analysis revealed that our prognostic model was an independent prognostic factor, capable of assessing patient prognosis autonomously, regardless of other clinical indicators (**Figure 4**). In addition, nomograms were constructed to evaluate the prognosis of ovarian cancer patients, and not only the calibration curves but also concordance index (C-index) results indicated that nomograms could accurately predict OS rates (**Figure 5A-C**). Additionally, the decision curve analysis (DCA) revealed that the risk score offered the greatest net benefit in predicting 5-year overall survival (OS) rates (**Figure 5D**). In summary, our risk model demonstrated extremely efficient predictive capability and held significant potential for clinical applications.

### Drug susceptibility prediction and tumor immune landscape

**Figure 6:**
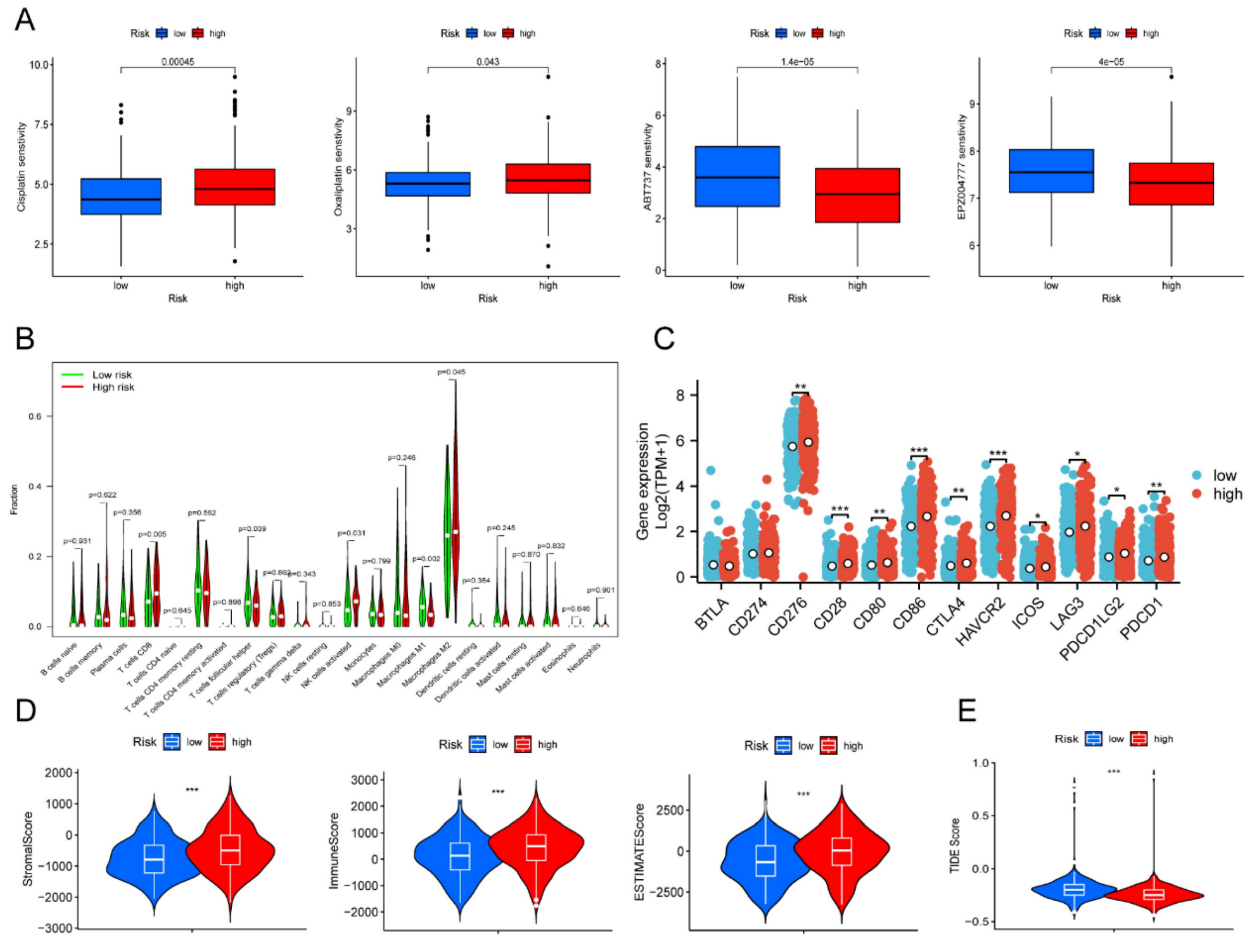
** Drug susceptibility prediction and tumor immune landscape.** (**A**) Drug sensitivity between the low- and high-risk patients. (**B**) Immune cell infiltration analysis between the low- and high-risk patients. (**C**) Differential expression of immune checkpoint genes between the low- and high-risk patients. (**D**) Violin plot displayed the stromal score, immune score, and ESTIMATE score between the low- and high-risk groups, respectively. (**E**) Violin plot revealed the TIDE score between the low- and high-risk groups. * *P* < 0.05, ** *P* < 0.01, ***P < 0.001.

To predict the response to small-molecule compounds and chemotherapeutics in the TCGA-OV cohort, we applied the "oncoPredict" R package to calculate the IC50 values. Our findings revealed that low-risk groups responded considerably more to cisplatin and oxaliplatin, while high-risk groups showed the opposite trend (**Figure 6A**). This suggested that platinum-based therapies might not be effective in high-risk patients. Our screening results revealed that 14 drugs exhibited greater treatment responsiveness in the high-risk group, including ABT737 (a Bcl2 inhibitor) and EPZ004777 (a disruptor of telomeric silencing 1-like inhibitor) (**Figure 6A, Figure S4**). Earlier research had demonstrated that ABT737 promoted apoptosis in OV cells by inhibiting aerobic glycolysis 30. In addition, the mechanism underlying EPZ004777 against OV involves the inhibition of amino acid and nucleotide biosynthesis pathways 31. Hence, personalized treatment guided by patient risk scores represents an encouraging treatment modality.

Next, we investigated whether immunotherapy could provide a potential therapeutic benefit for the high-risk group that exhibited poor response to platinum-based therapy. Due to the strong relationship between tumor metabolism and immunity 32, correlation analysis was performed to examine the relationship between different risk scores and immune infiltration levels in OV. The violin plot illustrated that the high-risk group showed higher levels of T cells CD8, NK cells, and M2 macrophages compared to the low-risk group (*P* < 0.05). On the other hand, the high-risk group presented lower levels of T cells follicular helper and macrophages M1 than the low-risk group (*P* < 0.05) (**Figure 6B**). Despite the higher enrichment of T cells CD8 and activated NK cells, the high-risk group still had an unfavorable prognosis in contrast to the low-risk group. We speculated the poor prognosis in the high-risk group was attributed to immune escape. As we expected, notable differences were discovered in immune checkpoint expression between the low- and high-risk groups. The high-risk group indicated increased expression levels of CD276, CD28, CD80, CTLA4, HAVCR2, ICOS, LAG3, PDCD1LG2, and PDCD1 compared to the low-risk group (**Figure 6C**). Survival analysis verified that elevated expression of multiple immune checkpoint genes, including CD28, CD276, CD80, CD86, CTLA4, HAVCR2, LAG3, PDCD1, and PDCD1LG2, was strongly associated with poor prognosis. Those results suggested that these immune checkpoint genes overexpression might be a crucial factor contributing to the poorer prognosis observed in high-risk patients (**Figure S5**). Furthermore, the results of our analysis of the tumor microenvironment revealed significantly greater stromal, immune, and ESTIMATE scores in the high-risk group than in the low-risk group (**Figure 6D**). In contrast, the TIDE score was extremely lower in the high-risk patient (**Figure 6E**). Based on the results above, our findings indicated that immunotherapy could represent a promising therapeutic approach for high-risk patients.

### Evaluation of Immunotherapy

**Figure 7:**
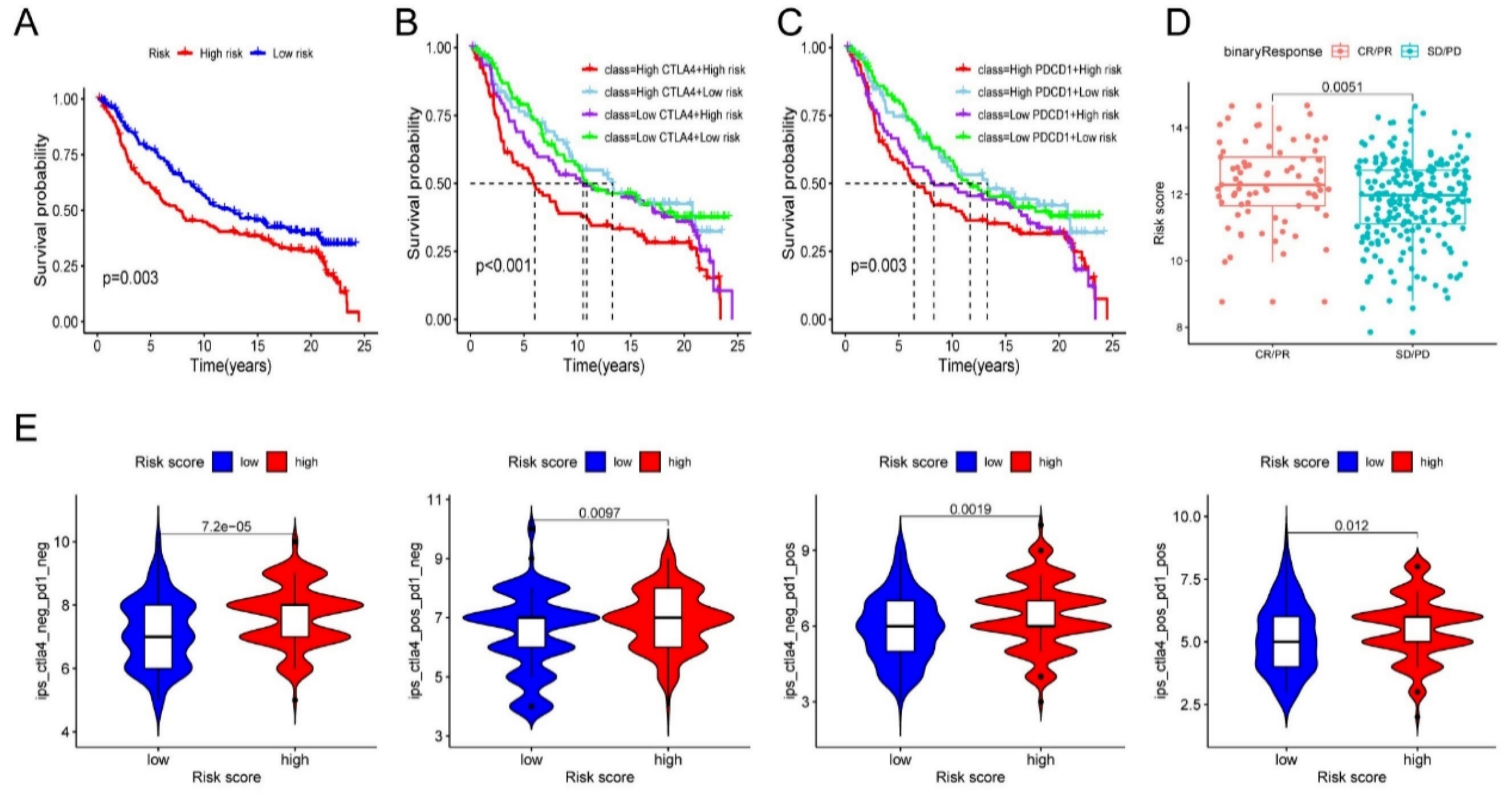
** Evaluation of immunotherapy. (A)** Survival analysis of IMvigor210 cohort established on risk model. (**B-C**) Survival analysis combined risk score with the expression of CTLA4 (**B**) or PDCD1 (**C**) in IMvigor210 cohort, respectively. (**D**) Immunotherapy response in diverse groups. (**E**) Evaluation of the IPS score to CTLA-4 and PD-1 between the low- and high-risk groups. * *P* < 0.05, ** *P* < 0.01, ***P < 0.001.

Given the above results, we evaluated whether immunotherapy could provide benefits to high-risk patients. We assessed the risk score for each individual sample in the IMvigor210 cohort based on the established risk model. Subsequently, all samples were classified into low- and high-risk groups based on the median risk score. The survival analysis demonstrated a significantly worse prognosis in high-risk patients (**Figure 7A**). And more importantly, we identified that high-risk samples with increased levels of CTLA4 or PDCD1 had the worst outcome (**Figure 7B-C**). Encouragingly, high-risk patients were more potential to benefit from ICB therapy and improve their outcome than the low-risk patients (**Figure 7D**). To validate the reaction of high-risk patients to immunotherapy, we conducted an analysis of the immune efficacy of targeting CTLA-4 and PD-1 using data from the TCIA dataset. The results were consistent with our prior study and provided confirmation that high-risk patients display a more favorable response to CTLA-4 and PD-1 immunotherapy in comparison to low-risk patients (**Figure 7E**). The above results indicated the clinical significance of the prognostic model in guiding immunotherapy.

### AAK1 promotes malignant progression of OV

**Figure 8:**
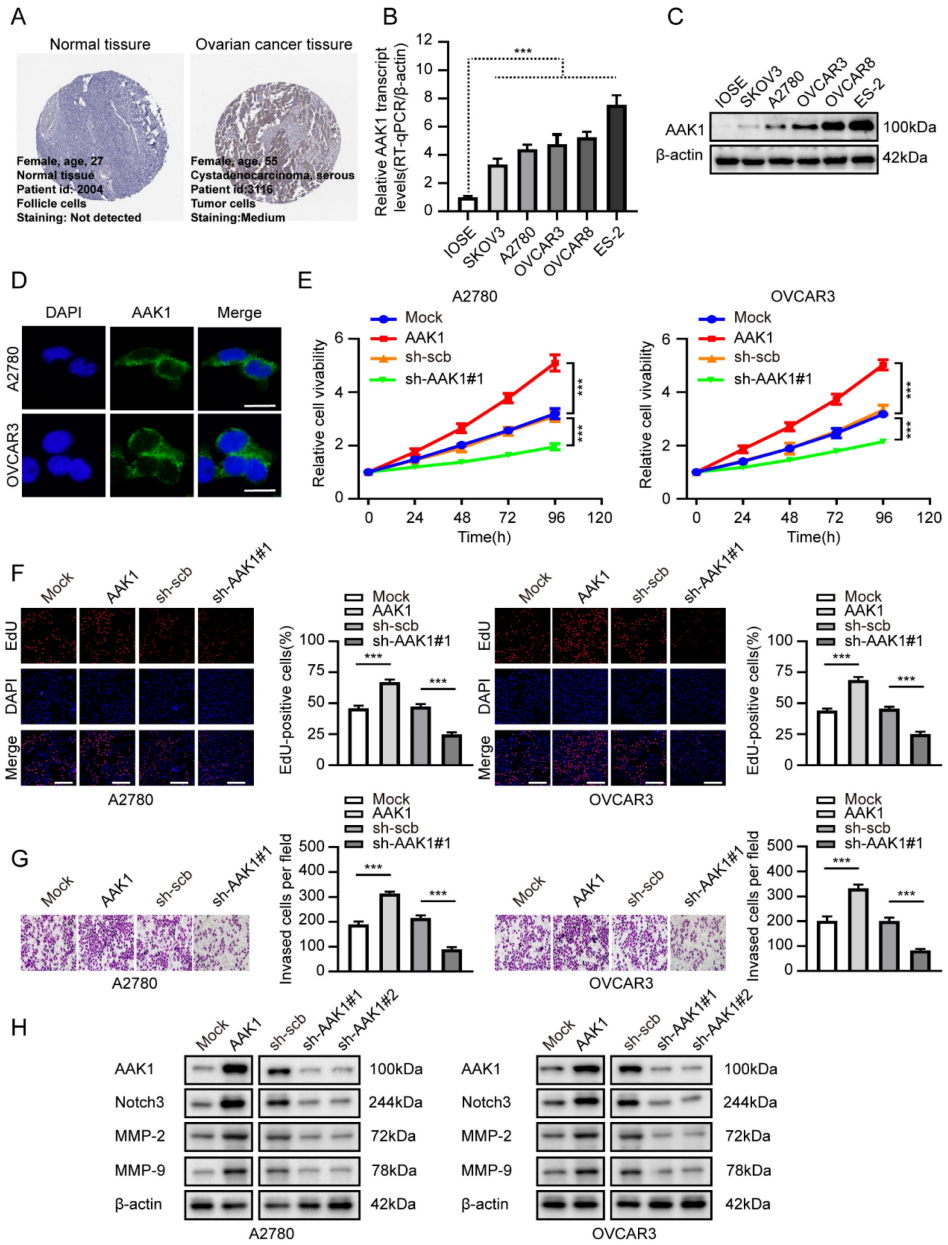
** AAK1 promotes OV progression via Notch3 pathway *in vitro*.** (**A**) Representative immunohistochemical staining images of AAK1 in normal ovary and OV tissue from The Human Protein Atlas (THPA) database. (**B-C**) Relative expression of AAK1 between IOSE-80 cell lines and five OV line were tested by RT-qPCR (**B**) (n = 3) and western blot (**C**) (n = 3), respectively. (**D**) Immunofluorescence staining showing the subcellular localization of AAK1 protein in A2780 and OVCAR3 cells (Scale bar: 10 µm). (**E-G**) CCK8 assay (**E**), Edu assays (**F,** Scale bar: 100 µm), and invasion assays (**G**) revealing the cell viability, proliferation, and invasion of A2780 and OVCAR3 cells stably transfected as indicated, respectively (n = 3). (**H**) Western blotting showing the protein levels of AAK1, Notch3, MMP-2, and MMP-9 in A2780 and OVCAR3 cells stably transfected as indicated, respectively (n = 3). * *P* < 0.05, ** *P* < 0.01, ***P < 0.001.

In our analysis of the gene expression and prognostic values of risk-model related genes, our findings revealed a consistent correlation between elevated expression levels of AAK1 and an unfavorable prognosis in OV patients. These observations provided evidence that AAK1 might play an oncogenic role in OV (**Figure S6A, Figure S6C**). Based on the consistency of its expression and poor prognosis, we selected AAK1 as our primary candidate for further investigation into its potential role in OV. Subsequently, we investigated the expression levels of AAK1 between OV and normal ovary tissues from The Human Protein Atlas (THPA, https://www.proteinatlas.org/) (**Figure 8A**). We also evaluated the mRNA and protein expression of AAK1 between IOSE-80 and OV cell lines. Results consistently demonstrated that AAK1 expression levels were significantly higher in both OV tissues as well as cancer cell lines when compared to normal tissues and cell lines (**Figure 8B-C**). Furthermore, immunofluorescence analysis indicated that AAK1 protein predominantly localizes to the cytoplasm (**Figure 8D**). We selected A2780 and OVCAR3 cell lines for follow-up investigations based on their endogenous expression levels of AAK1 (**Figure 8B-C**). Overexpression or knockdown of AAK1 resulted in the promotion or reduction of the cell viability, proliferation, and invasion in both A2780 and OVCAR3 cells, respectively (**Figure 8E-G**). While research on the role of AAK1 in tumor progression remains limited, previous study has suggested that AAK1 can stimulate the Notch pathway 33. Multiple studies have demonstrated that abnormal activation of the Notch pathway plays a crucial role in the malignant advancement of OV, and interventions targeting this pathway have shown promising anti-OV effects 34, 35.

The Notch protein family was comprised of four members: Notch1, Notch2, Notch3, and Notch4. We investigated the expression levels and survival significance of these four genes in OV based on the TCGA-OV database, and the results indicated that only Notch3 was markedly up-regulated and associated with poor prognosis in OV (**Figure S6B, Figure S6C**). A prior literature has suggested that Notch3 could facilitate the progression of liver cancer by upregulating MMP-2 and MMP-9 36. Furthermore, a high level of Notch3 expression indicated an unfavorable prognosis in liver cancer patients 36. Considering the previous exploration, we examined the AAK1 abnormal expression effect on Notch3-related pathway. As displayed in **Figure 8H**, elevating the expression of AAK1 resulted in an escalation of Notch3, MMP-2, and MMP-9 expression. Conversely, hindering AAK1 expression considerably diminished the expression of those molecules.

### AAK1 promotes glutamine metabolism of OV via Notch pathway

**Figure 9:**
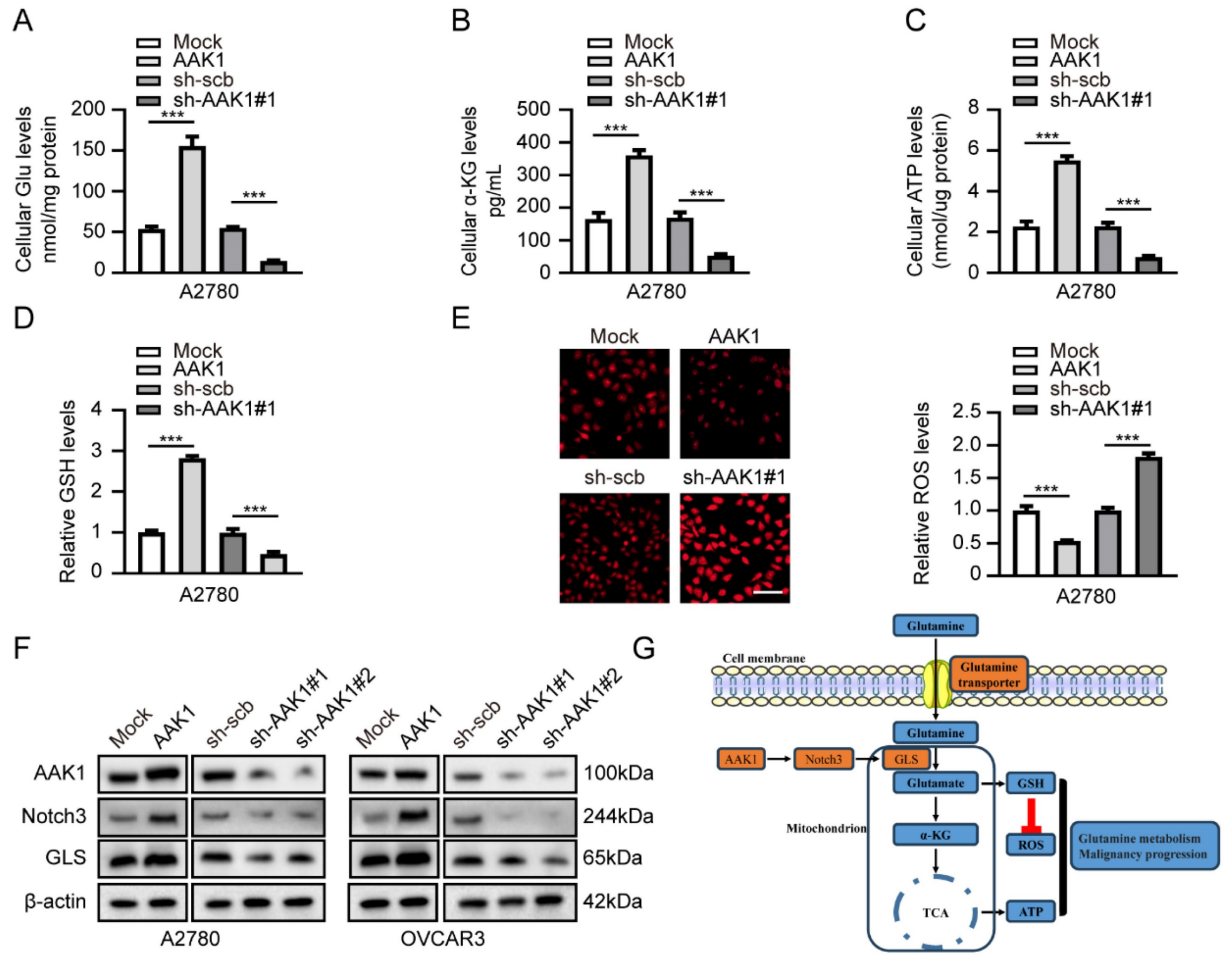
** AAK1 promotes glutamine metabolism of OV via Notch pathway in A2780 cells.** (**A-E**) The cellular Glu (**A**), α-KG (**B**), ATP (**C**), GSH (**D**), and ROS levels (E, Scale bar: 100 µm) of A2780 cells stably transfected as indicated (n = 3). (F) Western blot indicating the protein levels of AAK1, Notch3, and GLS in A2780 and OVCAR3 cells stably transfected as indicated (n = 3). (**G**) Schematic diagram of AAK1 promots glutamine metabolism and malignant progression through the Notch3 pathway in ovarian cancer. * *P* < 0.05, ** *P* < 0.01, ***P < 0.001.

Subsequently, we examined the potential role involvement of AAK1 in glutamine metabolism in OV. Glutamine metabolism is a crucial feature of tumor metabolic reprogramming, exerting significant effects on cancer cell biosynthesis, energy metabolism, and the maintenance of redox homeostasis 37. Hence, we examined various key indicators of glutamine metabolism in our experiment, including Glu, α-KG, ATP, GSH, and ROS. As expected, the overexpression of AAK1 resulted in elevated levels of glutamate, α-KG, ATP, and GSH, while reducing the levels of ROS in both A2780 and OVCAR3 cells (**Figure 9A-E, Figure S7A-E**). In contrast, the knockdown of AAK1 significantly inhibited the levels of Glu, α-KG, ATP, and GSH, while leading to an increase in ROS levels in both A2780 and OVCAR3 cells (**Figure 9A-E, Figure S7A-E**). Prior research showed that blocking the Notch pathway suppressed GLS expression and resulted in reducing intracellular glutamate levels in glioblastoma cells 38. GLS is a critical enzyme that controls the glutaminolysis pathway, which assumes a vital function in the conversion of glutamine to glutamate 15. Previous study had shown that highly expression of GLS indicated poor prognosis of OV patients 39. Considering the previous exploration, we examined the AAK1 abnormal expression effect on Notch3-GLS pathway. As displayed in **Figure 9F**, elevating the expression of AAK1 resulted in an escalation of Notch3 and GLS expression in A2780 and OVCAR3 cells. Conversely, hindering AAK1 expression considerably diminished the expression of Notch3 and GLS in A2780 and OVCAR3 cells. These results suggested that AAK1 promotes glutamine metabolism and malignant progression of OV via activating Notch3/GLS pathway.

### Therapeutic knockdown of AAK1 inhibits OV progression *in vivo*

**Figure 10:**
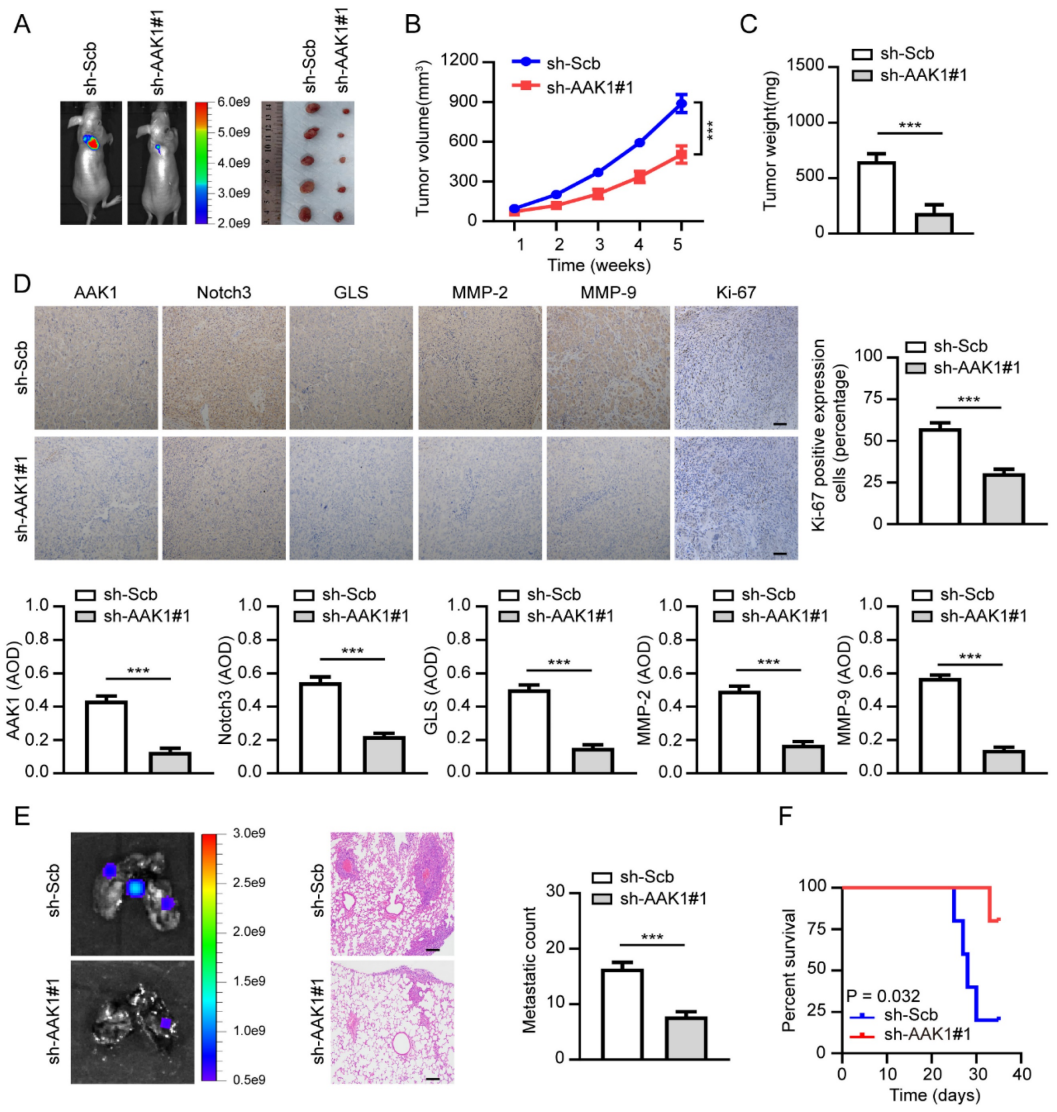
** Therapeutic knockdown of AAK1 suppresses OV progression and metastasis *in vivo*.** (**A**) The representative images, (**B**) tumor growth, (**C**) tumor weight, (**D**) the representative IHC images and statistical analysis results of AAK1, Notch3, GLS, MMP-2, MMP-9, and Ki-67 of xenograft tumors established by subcutaneous injection of OVCAR3 cells that were stably transfected as indicated, respectively, respectively (n = 5 per group). Scale bar: 50 μm. (**E**) The lung metastasis images (left panel) and metastatic counts (right panel) of tumor lung metastasis assay formed by tail vein injection of OVCAR3 cells that were stably transfected as indicated, respectively (n = 5 per group). Scale bar: 100 μm. (**G**) K-M curves were employed to display the survival of nude mice with distinct treatments in the tumor lung metastasis assay. * *P* < 0.05, ** *P* < 0.01, ***P < 0.001.

To further confirm the therapeutic potential of targeting AAK1 in OV *in vivo*, we created xenograft tumor models and tumor metastasis assays. As illustrated in **Figure 10A-F**, knockdown of AAK1 substantially suppressed tumor growth, tumor weight, as well as the expression of AKK1, Notch3, GLS, MMP-2, MMP-9, and Ki-67 percentage. Additionally, it resulted in decreased lung metastatic counts and extended survival. Collectively, these findings indicated that AAK1 drives the malignancy progression of OV by stimulating the Notch3 pathway, and AAK1 could represent an innovative and promising therapeutic target for OV patients.

## Discussion

Chemotherapy resistance and recurrence were the mainly determinants of poor prognosis in OV 40. Studies have revealed that metabolic reprogramming in OV was intimately associated with the malignant progression and chemotherapy resistance 13, 41. Many studies have verified different metabolic related prognostic models for OV to evaluate outcome and guide treatment 42, 43. However, considering the importance of glycolysis and glutamine metabolism in tumor progression and prognosis of OV patients, it is of great value to evaluate the prognosis of OV based on the differential genes between two metabolic subtypes. Unfortunately, there is still a lack of relevant research in OV.

In our analysis, we constructed a prognostic signature characterized by differentially expressed genes between glutaminolytic and glycolysis metabolic subtypes for the first time, which could effectively evaluate OV patient outcome. We selected eight AGGRGs (AAK1, GJB6, HMGN5, LPIN3, INTS6L, PPOX, SPAG4, and ZNF316) as the pertinent genes for building the risk gene signature. Previous study has confirmed that AAK1 could activate the Notch pathway 33, while Notch signaling deeply involved in OV invasion and metastasis, angiogenesis and chemotherapy resistance 44, 45. In addition, Notch signaling pathway was also implicated in glycolysis and glutaminolytic 46, 47. Our results suggested that AAK1 could promote the malignant progression and glutamine metabolism of OV by activating the Notch3 and GLS expression. Additionally, a recent study revealed that AAK1 could interact with MHC Class I molecules and inhibit cytotoxic T lymphocyte (CTL) response against respiratory syncytial virus (RSV) infection 48. On the other hand, increased glycolysis levels inhibited the MHC Class I protein levels in cancer cells 49. Those investigations indicated that the unfavorable prognosis of OV may be correlated with immune escape due to metabolic reprogramming on account of high AAK1 expression. GJB6 encoded one of the connexin proteins, which was an important biomarkers of invasion and metastasis in lung adenocarcinoma 50. This might be responsible for poor prognosis of OV patients with increasing GJB6 expression. Study had shown that HMGN5, a member of the high-mobility group N (HMGN) protein family, participated in malignant progression of various tumors 51. Regrettably, there has been no research shown to clarify the mechanism involved in HMGN5 regulated the OV progression until now. LPIN3 played a crucial role in regulating lipid metabolism, and variable splicing of LPIN3 regulates pyruvate and fatty acid metabolism in cervical cancer 52. However, the roles of LPIN3 in OV progression remains elusive. INTS6, as a tumor suppressor gene, could inhibit the malignant progression of certain cancers by down-regulating Wnt/β-catenin signaling 53, 54. Our study also found that INTS6 was a favorable prognosis gene for OV. SPAG4, was also defined as a novel potential cancer marker 55, however, the level of SPAG4 expression was decreased in glioma cells treated by glutamine deprivation 56. ZNF316 has been identified as a transcription factor, although its specific role in OV remains uncertain.

Study has shown that metabolic reprogramming was strongly correlated with tumor immune microenvironment and the efficacy of immunotherapy in OV 57. For instance, elevated uptake of glutamine by tumor cells led to limited uptake of glutamine by immune cells and resulted in immune escape by regulating PD-L1 expression 58. Aerobic glycolysis provided energy for malignant progression of tumor, while specific acid TME inhibited T cell function, leading to immune escape and promoting malignancy progression 59. These findings proposed that metabolic reprogramming, especially glutamine and glycolytic metabolism, played a crucial function in the modification of tumor microenvironment and immunotherapy response. In our investigation, we created a prognostic model established on differential expression genes between glutamine and glycolytic metabolism. Results have shown that up-regulated expression of several immune checkpoints is correlated to poor prognosis in high-risk OV patients. Additional analysis found that OV patients with elevated risk responded more effectively to immunotherapy compared to patients with low-risk. One research had demonstrated that combining glucose or glutamine metabolic pathway targeted therapy with PD-1/ PD-L1 checkpoint inhibition immunotherapy was a novel anti-tumor strategy 59. Our findings were beneficial in guiding the individualized treatment according to the prognosis model.

Additionally, to establish the efficacy of the AGGRGs signature, AAK1 was selected for function and mechanism validation. Our experiments confirmed that AAK1 expression was elevated in OV, and silencing of AAK1 decreased the cell viability, proliferation, invasion, and glutamine metabolism by inhibiting Notch3 signaling pathway. The results of *in vivo* experiments further supported the potential of targeting AAK1 as a viable therapeutic strategy for OV. In short, these findings further supported the validity of prognostic models based on genetic characteristics of glycolysis and glutamine metabolism.

However, there are several limitations to our study. The precise functions of model-related genes involved in the tumor metabolism and immunity of OV have not yet been extensively explored. Although the mechanism of AKK1 involved in the malignant progression in OV has been explored, other model-related genes remain needed further verification. Those limitations will be further investigated in our later research.

## Conclusions

In conclusion, OV patients were separated into four metabolic subtypes: quiescent type, glycolytic type, glutaminolytic type and mixed type. Notable differences existed in prognosis, tumor microenvironment, and response to immunotherapy across these subtypes. Our prognostic model based on glutaminolytic-glycolysis subtypes associated genes could accurately assess the survival outcome and immunotherapy response of OV patients. In a nutshell, our findings might be beneficial for assessing prognosis and guiding individualized therapy for OV patients.

## Supplementary Material

Supplementary figures and table.

## Figures and Tables

**Table 1:** Distribution of clinical characteristics across the four datasates

		TCGA-OV (N=420)		GSE26193 (N=107)		GSE63885 (N=75)		ICGC-OV (N=111)
Age								
≤60		231		NA		NA		66
>60		189		NA		NA		45
Grade								
G1		1		8		0		NA
G2		49		31		0		NA
G3		361		68		58		NA
G4		1		0		17		NA
Unknown		8		0		0		NA
Stage								
Ⅰ		1		20				0
Ⅱ		25		11		2		0
Ⅲ		328		59		63		96
Ⅳ		63		17		10		15
Unknown		3		0		0		0

## Abbreviations

AGGRGs: Aerobic glycolysis and glutamine-related genes
AUC: Area under the curve
BP: Biological process
CC: Cell composition
C-index: Concordance index
DCA: Decision curve analysis
DEGs: Differentially expressed genes
GLS: Glutaminase
Glu: Glutamate
GLUL: Glutamate-ammonia ligase
GO: Gene ontology
GS: Glutamine synthetase
GSH: Glutathione
HE: Hematoxylin and eosin
IHC: Immunohistochemistry
KEGG: Kyoto encyclopedia of genes and genomes
MF: Molecular function
MICU1: Mitochondrial calcium uptake 1
OD: Optical density
OS: Overall survival
OV: Ovarian cancer
ROS: Reactive oxygen species
TCGA: The cancer genome atlas
ICGC: International cancer genome consortium
TCIA: The cancer immunome atlas
THPA: The human protein atlas
TIDE: Tumor immune dysfunction and exclusion
WGCNA: Weighted gene co-expression network analysis
α-KG: α-Ketoglutaric acid
