# Supplementary material for: Comprehensive analysis based on glycolytic and glutaminolytic pathways signature for predicting prognosis and immunotherapy in ovarian cancer
Source: J Cancer. 2024 Jan 1;15(2):383–400. doi: 10.7150/jca.88359 (PMC10758027; doi:10.7150/jca.88359)
Supplement: Supplementary file 1 — Supplementary figures and table. [file jcav15p0383s1.pdf]

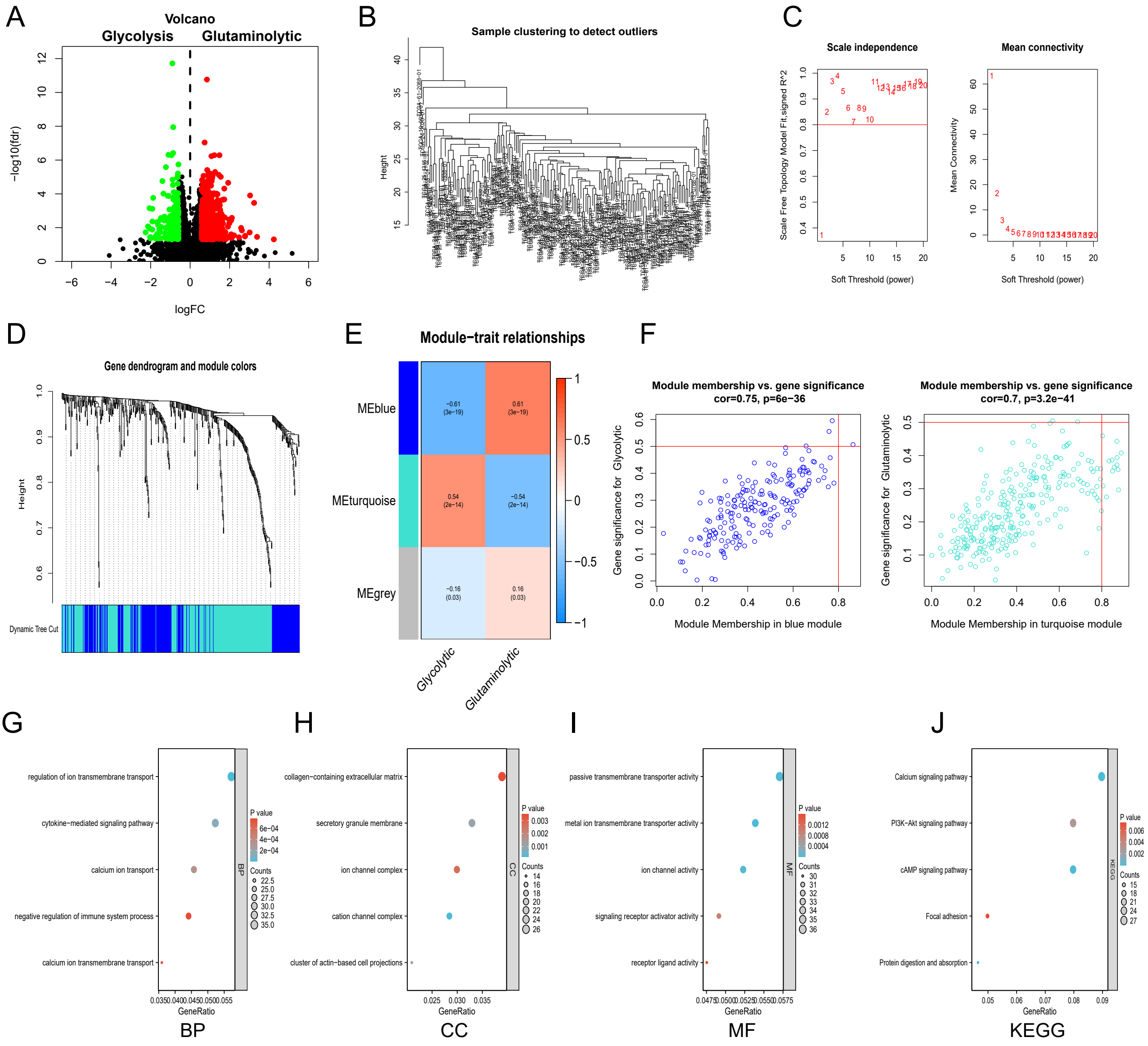

Figure S1 WGCNA to discover similar gene modules of AGGRGs. (A) The volcano map depicting the differentially expressed genes between glutaminolytic subtype and glycolytic subtype. (B) Cluster diagram of the TCGA-OV samples . (C) Identification of the optimal soft-threshold values. (D) Dendrogram of TCGA-OV gene clustering. (E) Heatmap showing the correlation between gene modules and glycolysis-glutaminolytic subtypes. (F) Scatter plot displaying the correlation between blue (right panel) and turquoise (left panel) module and gene significance, respectively. (G-J) The GO (G-I) and KEGG (J) enrichment analysis based on the dysregulated genes between the glutaminolytic type and glycolytic type.

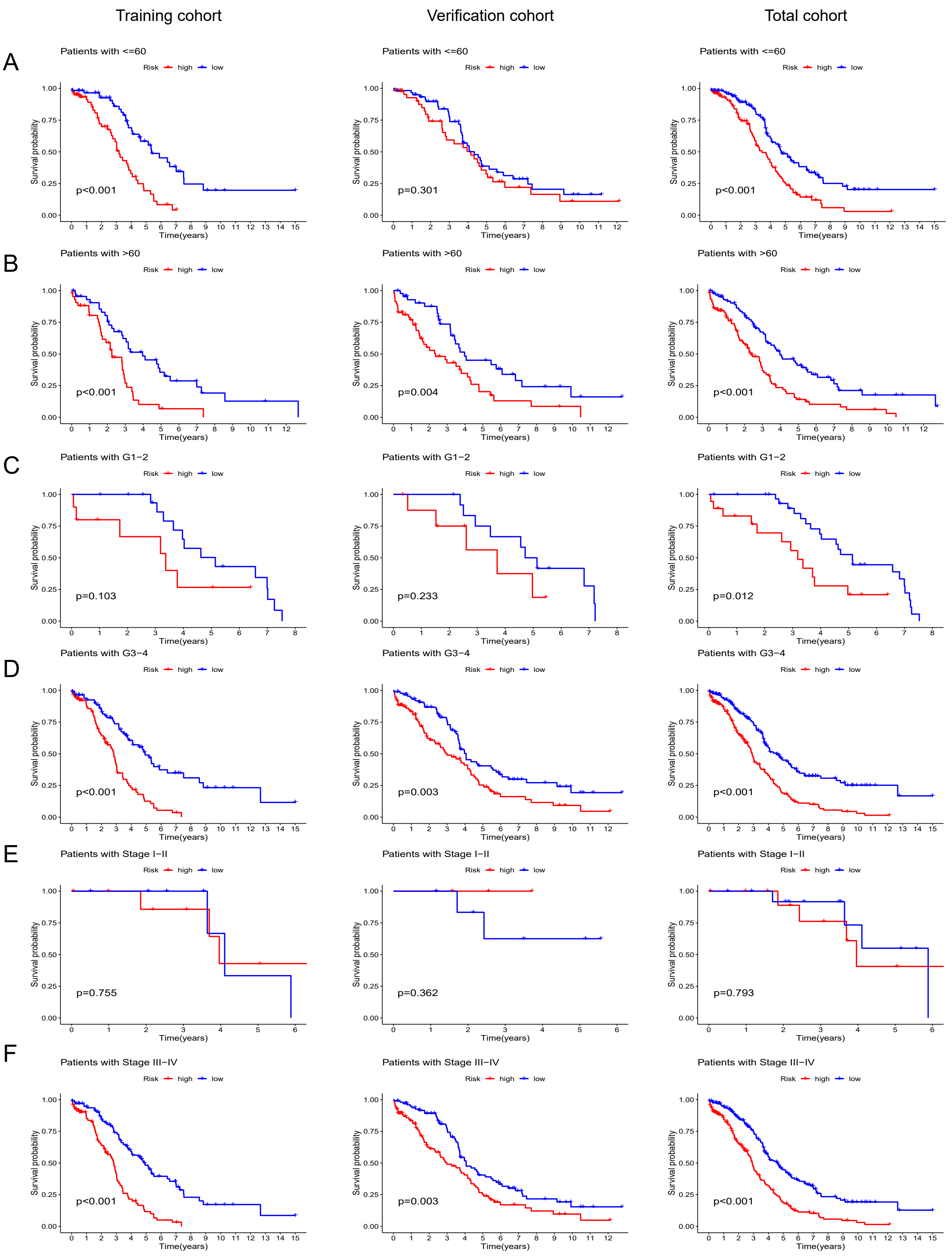

Figure S2 Prognostic role of the prognostic signature in clinical subgroups. (A-F) Prognostic analysis in age (A-B), grade (C-D), and stage (E-F) subgroups based on AGGRGs model in training, verification, and total cohorts, respectively.

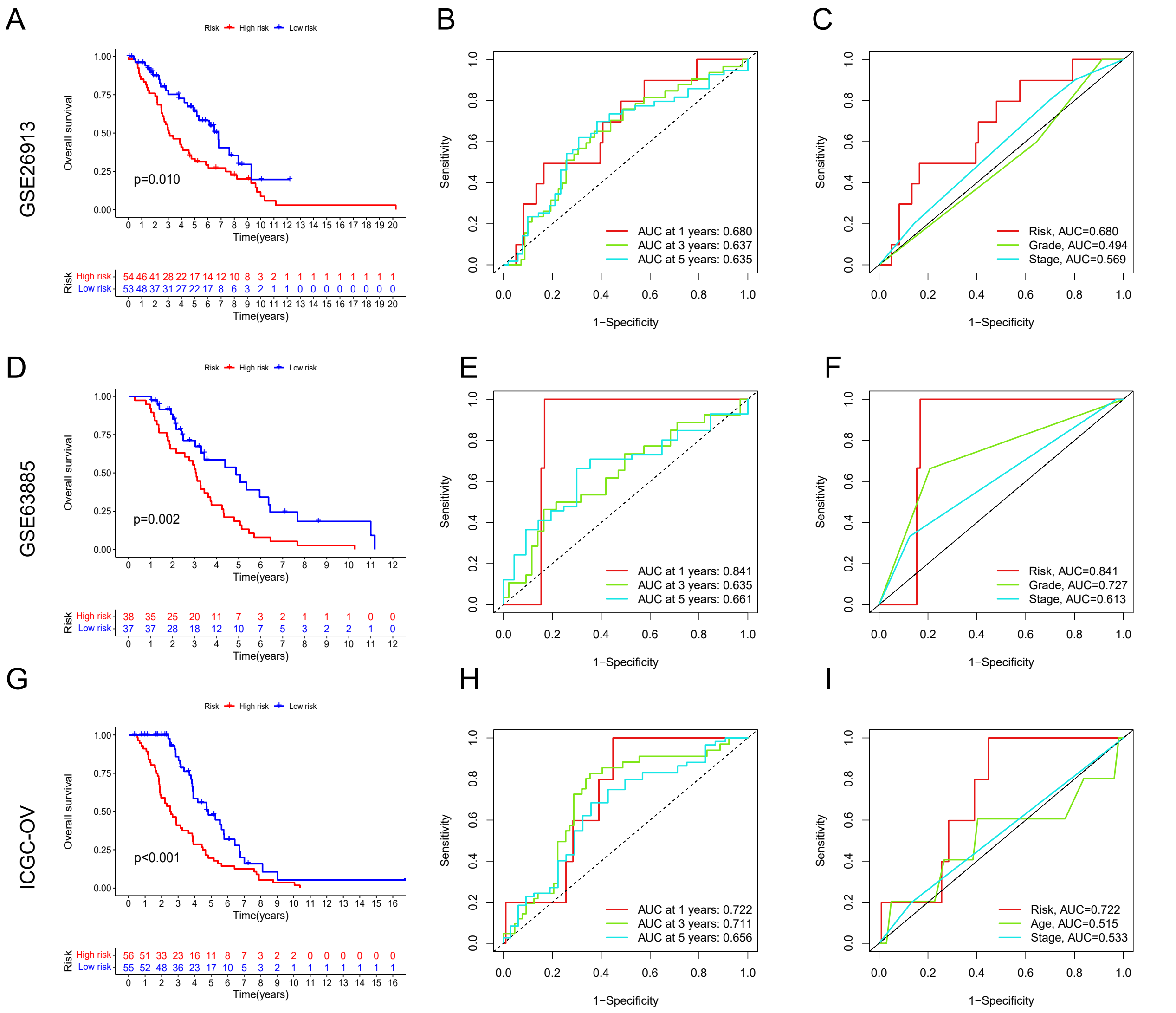

Figure S3 External validation of the AGGRGs risk prognostic model. (A-C) Represent the K-M plot (A), ROC curves for the prognostic value (B), and ROC curves comparing the prognostic ability of the risk score with other clinical indices for OS (C) in GSE26913, respectively. (D-F) Represent the K-M plot (D), ROC curves for the prognostic value (E), and ROC curves comparing the prognostic ability of the risk score with other clinical indices for OS (F) in GSE26913, respectively. (G-I) Represent the K-M plot (D), ROC curves for the prognostic value (E), and ROC curves comparing the prognostic ability of the risk score with other clinical indices for OS (F) in ICGC-OV, respectively.

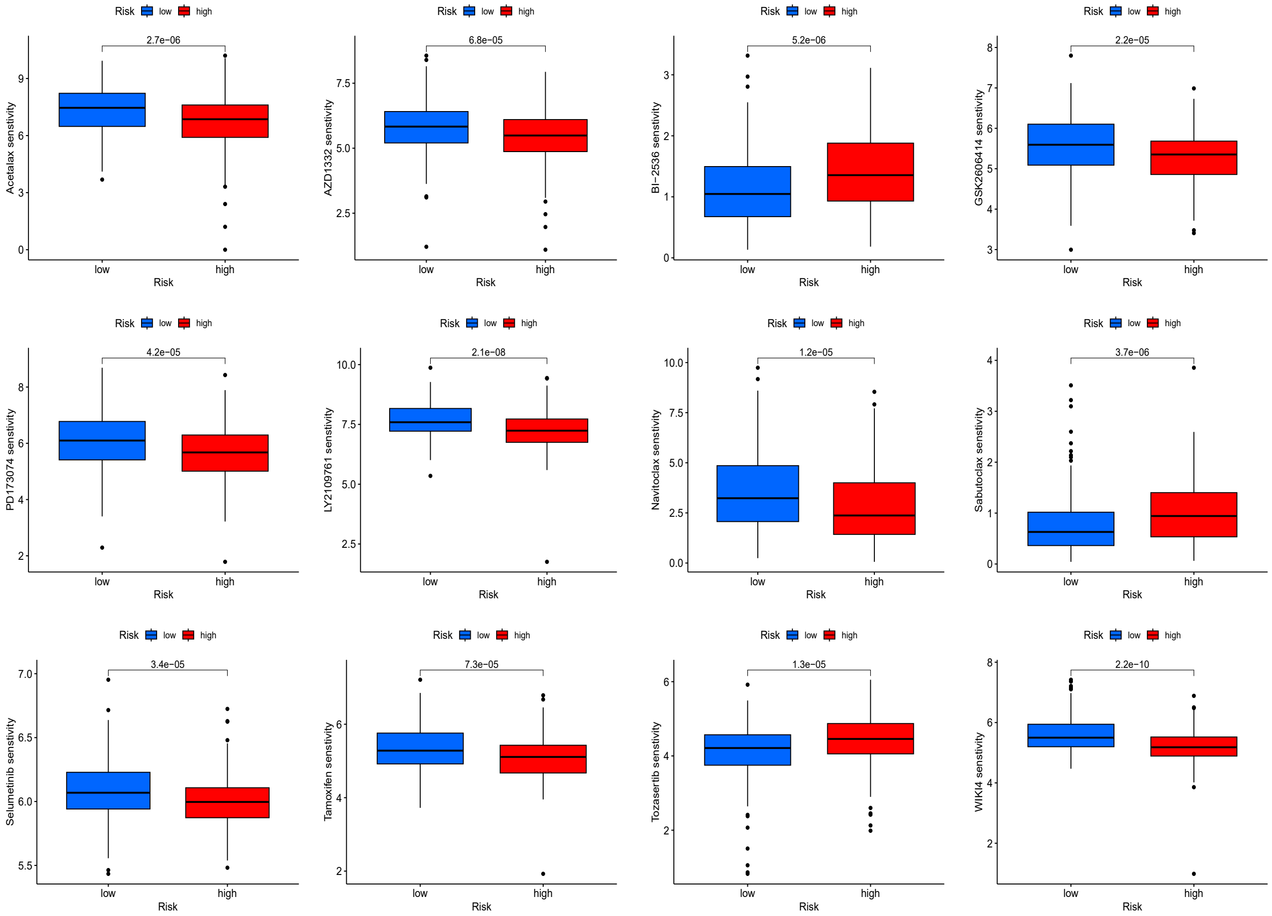

Figure S4 Drug susceptibility prediction based on risk model.

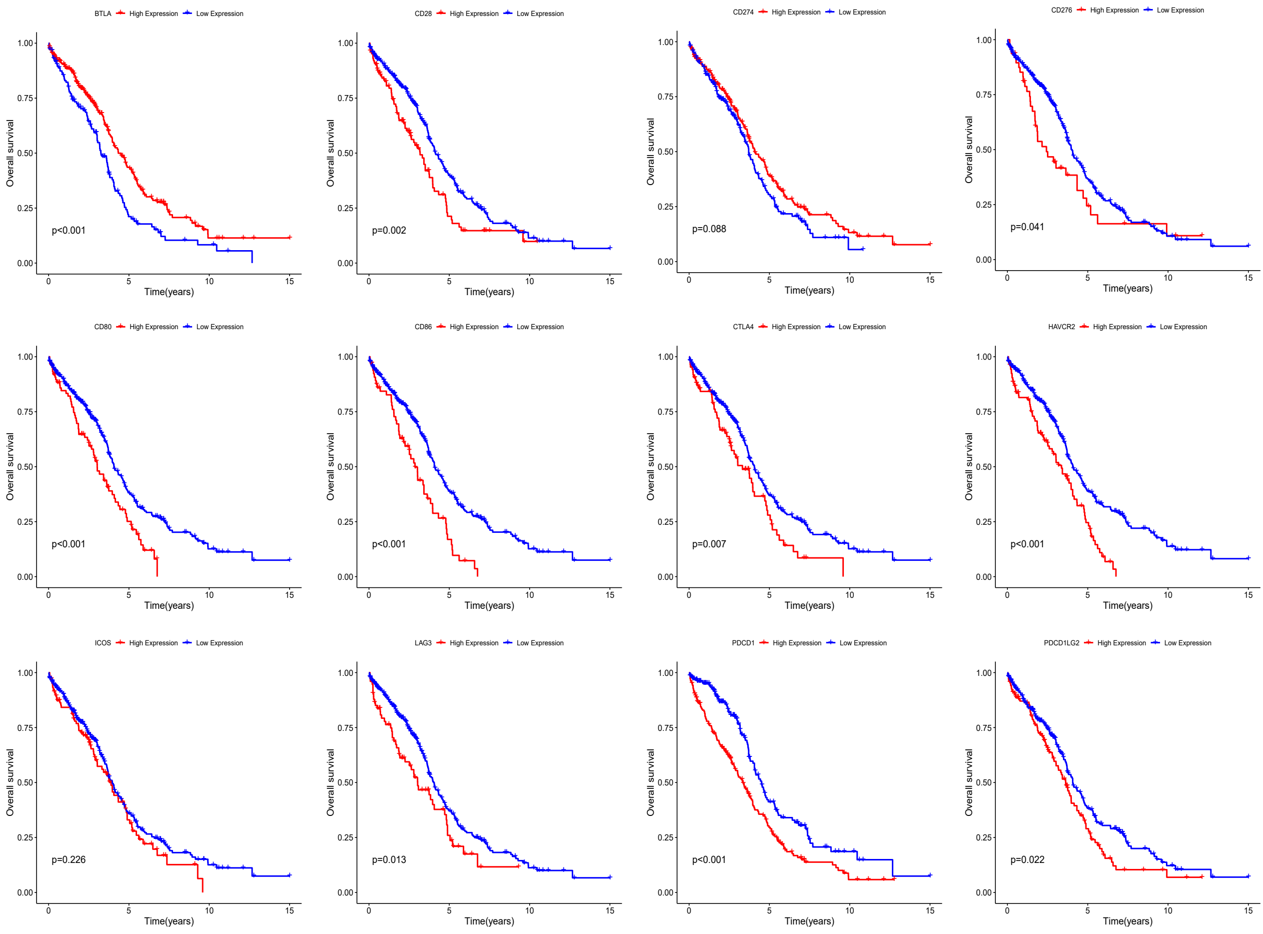

Figure S5 Survival curve of the immune checkpoint genes.

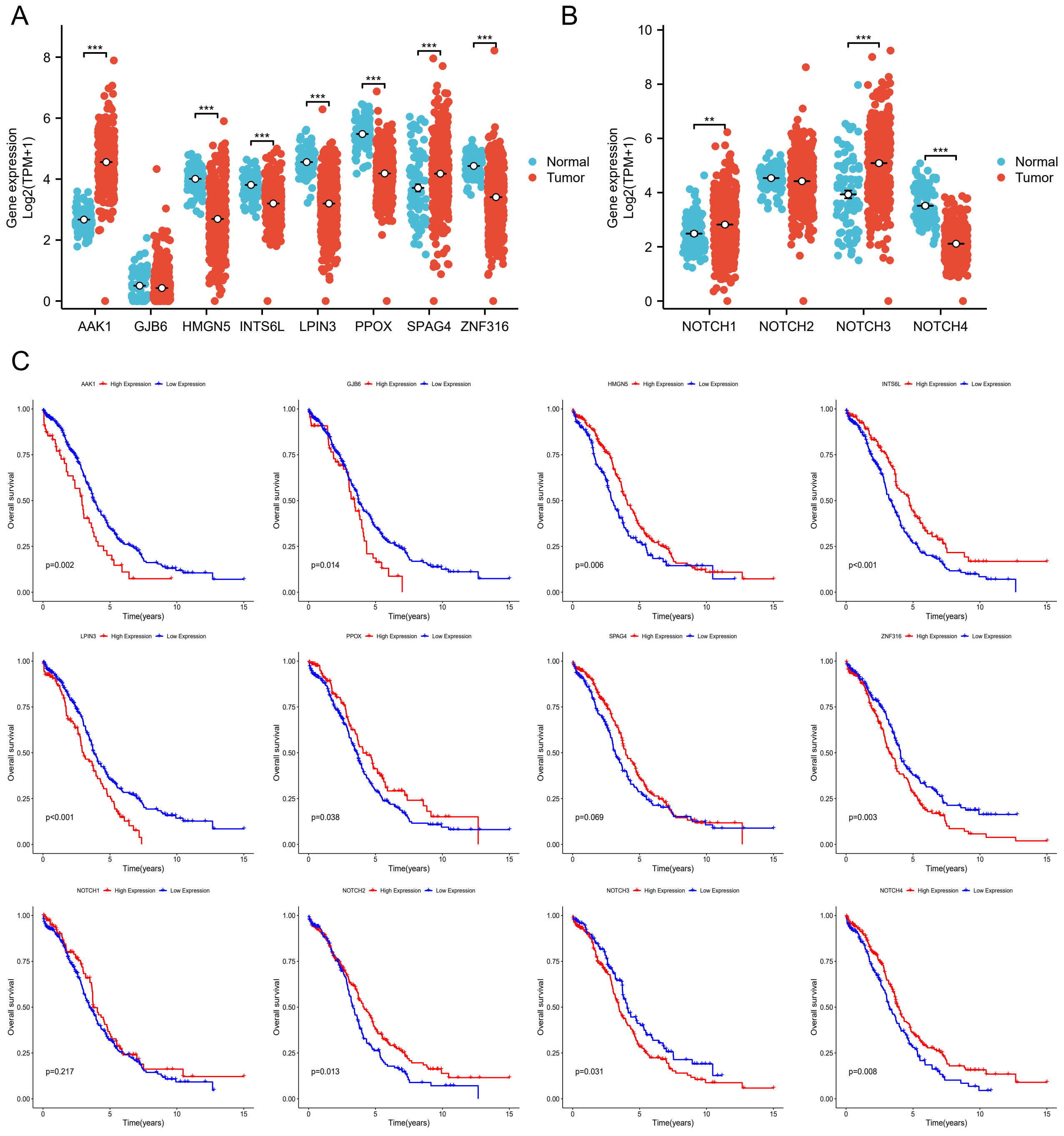

Figure S6 The expression level and survival curve of risk model-related genes and Notch family genes. (A-B) The expression level of risk model-related genes (A) and Notch family genes (B) between the low- and high-risk groups, respectively. (C) Survival curve of risk model-related genes and Notch family genes.

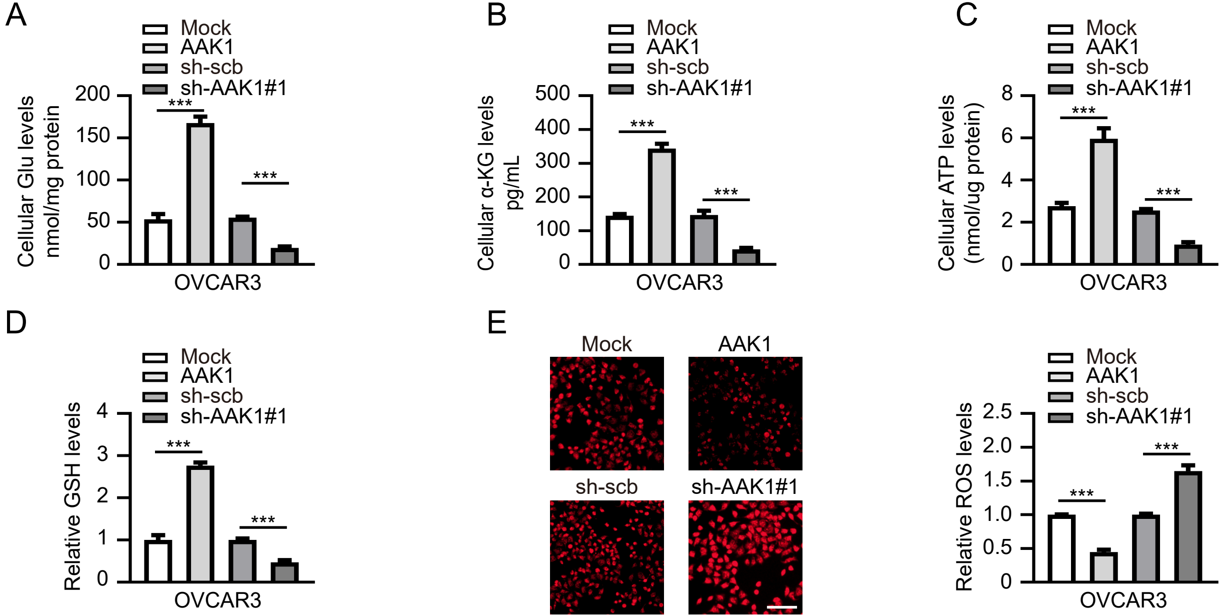

Figure S7 AAK1 promotes glutamine metabolism of OV via Notch pathway in OVCAR3 cells. (A-E) The cellular Glu (A), α-KG (B), ATP (C), GSH (D), and ROS levels (E, Scale bar: 100 μm) of OVCAR3 cells stably transfected as indicated (n = 3). * $P < 0.05$, ** $P < 0.01$, ***$P < 0.001$.

Table S1 The 12 glycolytic and 27 glutaminolytic genes were collected from the MSigDB database, respectively.

WP_AEROBIC_GLYCOLYSIS, n = 12	GOBP_GLUTAMINE_FAMILY_AMINO_ACID_CATABOLIC_PROCESS, n = 27
ALDOA	ADHFE1
ENO1	ALDH4A1
GAPDH	ARG1
GPI	ARG2
HK1	ARHGAP11B
LDHA	ASRGL1
PFKM	ATP2B4
PGAM2	DAO
PGK1	DDAH1
PKM	DDAH2
SLC2A1	FAH
TPI1	GAD1
	GAD2
	GLS
	GLS2
	GLUD1
	GLUD2
	GLUL
	GOT1
	GOT2
	MIR21
	NOS1
	NOS2
	NOS3
